# Supplementary material for: The Systems Biology Research Tool: evolvable open-source software
Source: BMC Syst Biol. 2008 Jun 29;2:55. doi: 10.1186/1752-0509-2-55 (PMC2446383; doi:10.1186/1752-0509-2-55)
Supplement: Additional file 1 — SBRT Archive. An archive of the current version of the Systems Biology Research Tool. [file 1752-0509-2-55-S1.zip › sbrt-1.4.0/doc/developers_guide/api/sbrt/shell/text/fba/FluxCapFormatV1.html]

FluxCapFormatV1


|  |  |  |
| --- | --- | --- |
| **Overview** \| **Package** \| **Class** \| **Use** \| **Tree** \| **Deprecated** \| **Index** \| **Help** | |  |
| **PREV CLASS**   **NEXT CLASS** | **FRAMES**    **NO FRAMES**     **All Classes** |  |
| SUMMARY: NESTED \| FIELD \| CONSTR \| METHOD | DETAIL: FIELD \| CONSTR \| METHOD |  |


---


## sbrt.shell.text.fba Class FluxCapFormatV1

```
java.lang.Object
  sbrt.shell.text.AbstractMapFormat<java.lang.String,java.util.Set<LinearComb<java.lang.String>>>
      sbrt.shell.text.fba.FluxCapFormatV1
```

**All Implemented Interfaces:**: FluxCapFormat, Format, MapFormat<java.lang.String,java.util.Set<LinearComb<java.lang.String>>>

---

``` public class FluxCapFormatV1 extends AbstractMapFormat<java.lang.String,java.util.Set<LinearComb<java.lang.String>>> implements FluxCapFormat ```

This class is a concrete implementation of `FluxCapFormat`.

**Author:**
:   This class was written and documented by
    Jeremiah Wright while in the Wagner lab.

---

**Constructor Summary**

`FluxCapFormatV1(Fluxome fluxome)`
  
          Constructs a new flux cap file line format using the
provided fluxome.


| **Method Summary** | |
| --- | --- |
| `Fluxome` | `getFluxome()`             Returns the fluxome used to verify reaction names. |
| `PipeListFormat<java.util.Set<LinearComb<java.lang.String>>>` | `getListFormat()`             Returns the format used for lists of values. |
| `ColonMapEntryFormatV1<java.lang.String,java.lang.String>` | `getMapEntryFormat()`             Returns the format used for variable-value pairs. |
| `PipeSetFormat<java.lang.String>` | `getSetFormat()`             Returns the format used for sets of variables. |

**Methods inherited from class sbrt.shell.text.AbstractMapFormat**

`formatKey, formatKeys, formatPair, formatPair, formatValue, formatValues, formatValues, getKeyFormatter, getKeyParser, getValueFormatter, getValueParser, parseAllKeys, parseAllPairs, parseAllValues, parseAllValues, parseKey, parsePair, parseValue`

**Methods inherited from class java.lang.Object**

`clone, equals, finalize, getClass, hashCode, notify, notifyAll, toString, wait, wait, wait`

**Methods inherited from interface sbrt.shell.text.MapFormat**

`formatKey, formatKeys, formatPair, formatPair, formatValue, formatValues, formatValues, parseAllKeys, parseAllPairs, parseAllValues, parseAllValues, parseKey, parsePair, parseValue`

**Constructor Detail**

### FluxCapFormatV1

```
public FluxCapFormatV1(Fluxome fluxome)
```

:   Constructs a new flux cap file line format using the
    provided fluxome.

    **Parameters:**: `fluxome` - the fluxome with which to verify reaction names. **Throws:**: `java.lang.NullPointerException` - if the provided argument is `null`.


**Method Detail**

### getFluxome

```
public Fluxome getFluxome()
```

:   Returns the fluxome used to verify reaction names.

    :   **Specified by:**: `getFluxome` in interface `FluxCapFormat`
    :   **Returns:**: the fluxome used to verify reaction names.

---


### getMapEntryFormat

```
public ColonMapEntryFormatV1<java.lang.String,java.lang.String> getMapEntryFormat()
```

:   Returns the format used for variable-value pairs.

    :   **Specified by:**: `getMapEntryFormat` in class `AbstractMapFormat<java.lang.String,java.util.Set<LinearComb<java.lang.String>>>`
    :   **Returns:**: the format used for variable-value pairs.

---


### getSetFormat

```
public PipeSetFormat<java.lang.String> getSetFormat()
```

:   Returns the format used for sets of variables.

    :   **Specified by:**: `getSetFormat` in class `AbstractMapFormat<java.lang.String,java.util.Set<LinearComb<java.lang.String>>>`
    :   **Returns:**: the format used for sets of variables.

---


### getListFormat

```
public PipeListFormat<java.util.Set<LinearComb<java.lang.String>>> getListFormat()
```

:   Returns the format used for lists of values.

    :   **Specified by:**: `getListFormat` in class `AbstractMapFormat<java.lang.String,java.util.Set<LinearComb<java.lang.String>>>`
    :   **Returns:**: the format used for lists of values.


---


|  |  |  |
| --- | --- | --- |
| **Overview** \| **Package** \| **Class** \| **Use** \| **Tree** \| **Deprecated** \| **Index** \| **Help** | |  |
| **PREV CLASS**   **NEXT CLASS** | **FRAMES**    **NO FRAMES**     **All Classes** |  |
| SUMMARY: NESTED \| FIELD \| CONSTR \| METHOD | DETAIL: FIELD \| CONSTR \| METHOD |  |


---
